# The Hidden Hazard of Hypothyroidism: Statin-Associated Rhabdomyolysis With Life-Threatening Complications

**DOI:** 10.7759/cureus.85506

**Published:** 2025-06-07

**Authors:** George Bechir, Angelina Bechir

**Affiliations:** 1 Hospital Medicine, Franciscan Health Munster, Munster, USA; 2 Genetics, Clemson University, Clemson, USA

**Keywords:** acute kidney injury, dialysis, hypothyroidism, muscle enzyme elevation, rhabdomyolysis, statin-associated myopathy, statin toxicity, thyroid dysfunction, transaminitis, uncontrolled hypothyroidism

## Abstract

Statins are among the most widely prescribed medications for reducing cardiovascular morbidity and mortality. While generally well tolerated, they carry a rare but potentially fatal risk of rhabdomyolysis - a condition marked by massive skeletal muscle breakdown, electrolyte abnormalities, and acute kidney injury (AKI). We report the case of a 76-year-old man who developed profound rhabdomyolysis, severe transaminitis, and dialysis, requiring AKI shortly after initiating atorvastatin. His initial symptoms included excruciating bilateral lower extremity pain and progressive weakness, ultimately rendering him unable to walk. Laboratory evaluation revealed a creatine kinase level exceeding 25,000 U/L, marked elevations in aspartate aminotransferase (AST) and alanine aminotransferase (ALT), and a creatinine of 9.6 mg/dL. Despite prompt initiation of aggressive intravenous hydration, bladder decompression, and supportive care, the patient remained anuric and progressed to volume overload, necessitating initiation of hemodialysis.

Further evaluation revealed profoundly uncontrolled hypothyroidism, with a thyroid-stimulating hormone (TSH) of 121.7 µIU/mL and a free T4 level of less than 0.4 ng/dL, despite reported use of levothyroxine. MRI of the thigh showed diffuse muscular edema without myonecrosis, and an autoimmune myositis panel was negative. Atorvastatin was promptly discontinued, and thyroid hormone therapy was intensified. Over the next several days, his biochemical parameters slowly improved, and he was ultimately discharged to acute rehabilitation with plans for outpatient dialysis and endocrine follow-up.

This case underscores the synergistic danger of statin therapy in the presence of untreated or undertreated hypothyroidism. Routine screening for thyroid dysfunction, particularly in elderly patients or those with known thyroid disease, may help prevent catastrophic complications such as rhabdomyolysis and irreversible renal injury.

## Introduction

Statins are among the most widely prescribed medications worldwide due to their proven benefit in reducing cardiovascular morbidity and mortality. Although generally well tolerated, statins carry a small but clinically significant risk of rhabdomyolysis, particularly when additional risk factors are present [1]. This potentially life-threatening condition, defined by skeletal muscle breakdown and marked elevation in creatine kinase (CK), can lead to acute kidney injury (AKI), electrolyte imbalances, and in severe cases, dialysis or intensive care admission [2].

While the myotoxic potential of statins is well recognized, the compounding effect of hypothyroidism is underappreciated. Even in subclinical or asymptomatic forms, hypothyroidism may increase the risk of statin-induced muscle injury by impairing muscle oxidative metabolism, weakening membrane integrity, and potentially reducing hepatic statin clearance via cytochrome P450 downregulation [3]. This is particularly relevant in elderly patients, among whom both hypothyroidism and statin use are highly prevalent. Several case reports have demonstrated that patients with untreated or under-replaced hypothyroidism may develop profound rhabdomyolysis and AKI shortly after starting statin therapy [4-7].

Additionally, transaminitis can occur in statin-induced rhabdomyolysis due to muscle-derived enzyme release, potentially mimicking hepatocellular injury. This overlap may delay diagnosis and mislead clinicians, particularly in the absence of overt myopathic symptoms [8].

Here, we present the case of a 76-year-old man who developed severe rhabdomyolysis, transaminitis, and dialysis-dependent AKI following initiation of atorvastatin. Notably, he was found to have markedly uncontrolled hypothyroidism despite being on thyroid replacement therapy. This case highlights the need for heightened clinical vigilance, and while formal guidelines do not recommend routine thyroid screening prior to statin therapy, our case and others support considering thyroid function testing in older adults and patients with suspected or known thyroid disease.

## Case presentation

A 76-year-old male with a medical history of hypertension, type 2 diabetes mellitus, coronary artery disease (status post-stent), peripheral vascular disease, and hypothyroidism presented to the emergency department with progressive bilateral lower extremity pain and inability to walk. He described the pain as excruciating, constant, and worsening over the past week, without preceding trauma, strenuous activity, illness, or fall. He had undergone lower extremity stenting one year prior, but denied recent vascular symptoms.

Two weeks before admission, the patient had been started on atorvastatin for secondary cardiovascular prevention. He reported adherence to his home medications, including levothyroxine, but had not had recent thyroid function testing. On examination, he appeared fatigued and weak, with marked tenderness over both thighs but no focal neurologic deficits or signs of compartment syndrome. He was afebrile and hemodynamically stable. Peripheral pulses were intact.

Initial laboratory testing revealed significant abnormalities: creatinine 9.6 mg/dL, blood urea nitrogen (BUN) 84 mg/dL, aspartate aminotransferase (AST) 968 U/L, alanine aminotransferase 498 U/L, and alkaline phosphatase 540 U/L, consistent with AKI and transaminitis. Total bilirubin remained within normal limits. Despite aggressive intravenous hydration, bladder decompression, and Foley catheter placement, the patient remained anuric. Laboratory findings at presentation, including severe hypothyroidism, rhabdomyolysis, and metabolic derangements, are summarized in Table 1.

**Table 1:** Laboratory Findings on Admission (04/17/2025) BUN: blood urea nitrogen; eGFR: estimated glomerular filtration rate; AST: aspartate aminotransferase; ALT: alanine aminotransferase; TSH: thyroid-stimulating hormone; T4: free thyroxine; HbA1c: hemoglobin A1c; CBC: complete blood count; WBC: white blood cells; SGOT: serum glutamic-oxaloacetic transaminase; SGPT: serum glutamic-pyruvic transaminase

Laboratory Test	Result	Reference Range	Interpretation
Electrolytes and Renal Function			
Sodium (Na)	132 mmol/L	135-145 mmol/L	Low
Potassium (K)	4.8 mmol/L	3.5-5.0 mmol/L	Normal
Chloride (Cl)	96 mmol/L	98-107 mmol/L	Low
Bicarbonate (CO_2_)	17.5 mmol/L	22-29 mmol/L	Low
Anion Gap	18.5	8-16	High
BUN	84 mg/dL	7-20 mg/dL	High
Creatinine	9.6 mg/dL	0.6-1.3 mg/dL	High
eGFR	5 mL/min/1.73m^2^	>60 mL/min/1.73m^2^	Critically Low
Calcium	6.5 mg/dL	8.5-10.5 mg/dL	Low
Magnesium	2.6 mg/dL	1.7-2.3 mg/dL	Mildly High
Glucose (random)	122 mg/dL	70-100 mg/dL (fasting)	Mildly High
Liver Function			
AST (SGOT)	968 U/L	10-40 U/L	Critically High
ALT (SGPT)	498 U/L	7-56 U/L	Critically High
Alkaline Phosphatase	540 U/L	44-147 U/L	High
Bilirubin, Total	0.6 mg/dL	0.2-1.2 mg/dL	Normal
Albumin	2.8 g/dL	3.5-5.0 g/dL	Low
Total Protein	5.3 g/dL	6.4-8.3 g/dL	Low
Muscle Injury Markers			
CK (Creatine Kinase), Total	>20,000 U/L	20-200 U/L	Critically High
Thyroid Function			
TSH	121.7 µIU/mL	0.4-4.0 µIU/mL	Critically High
Free T4	<0.4 ng/dL	0.9-1.7 ng/dL	Critically Low
Diabetes Control			
HbA1c	8.6%	<5.7% (normal), <7% (goal)	High
CBC			
WBC	16.5 x10^3^/µL	4.5-11.0 x10^3^/µL	High
Hemoglobin	14.3 g/dL	13.5-17.5 g/dL	Normal
Platelets	440 x10^3^/µL	150-400 x10^3^/µL	Mildly High

A CK level obtained due to ongoing muscle pain was >25,000 U/L, exceeding the assay’s upper detection limit, and remained at this level for nearly 10 consecutive days. Urinalysis revealed 3+ blood, 2+ protein, and pyuria. Urine culture grew *Enterococcus*, and broad-spectrum antibiotics were initiated for a suspected complicated urinary tract infection.

**Figure 1:**
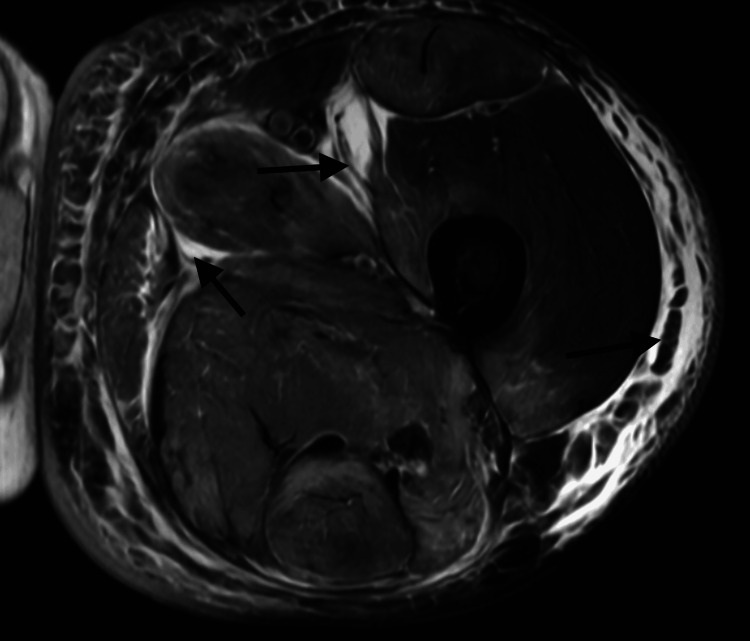
Axial T2-Weighted MRI of the Left Thigh The MRI shows a diffuse hyperintense signal throughout the thigh musculature (black arrows), consistent with extensive muscular edema. There is also subcutaneous and interfascial edema, without evidence of myonecrosis or abscess formation. These findings are characteristic of severe myositis or rhabdomyolysis.

**Figure 2:**
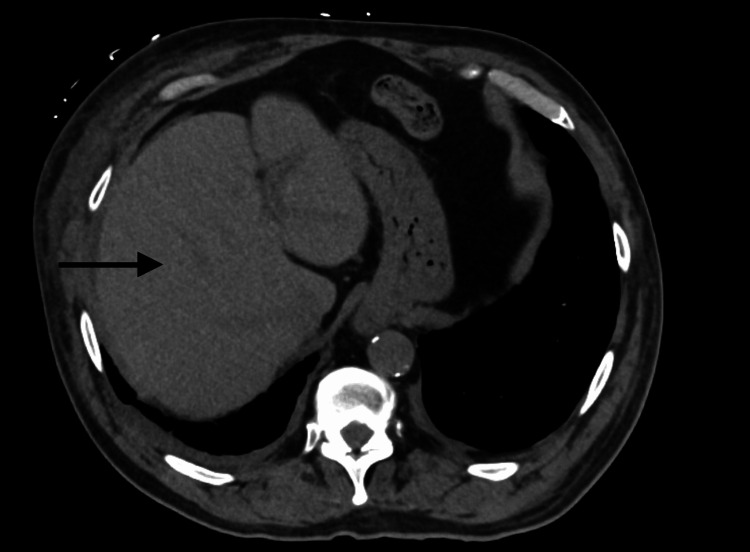
Axial Non-contrast CT of the Abdomen The axial non-contrast CT image shows the liver with preserved parenchymal architecture and no evidence of biliary dilation, obstructive pathology, or focal hepatic lesions (black arrow).

**Figure 3:**
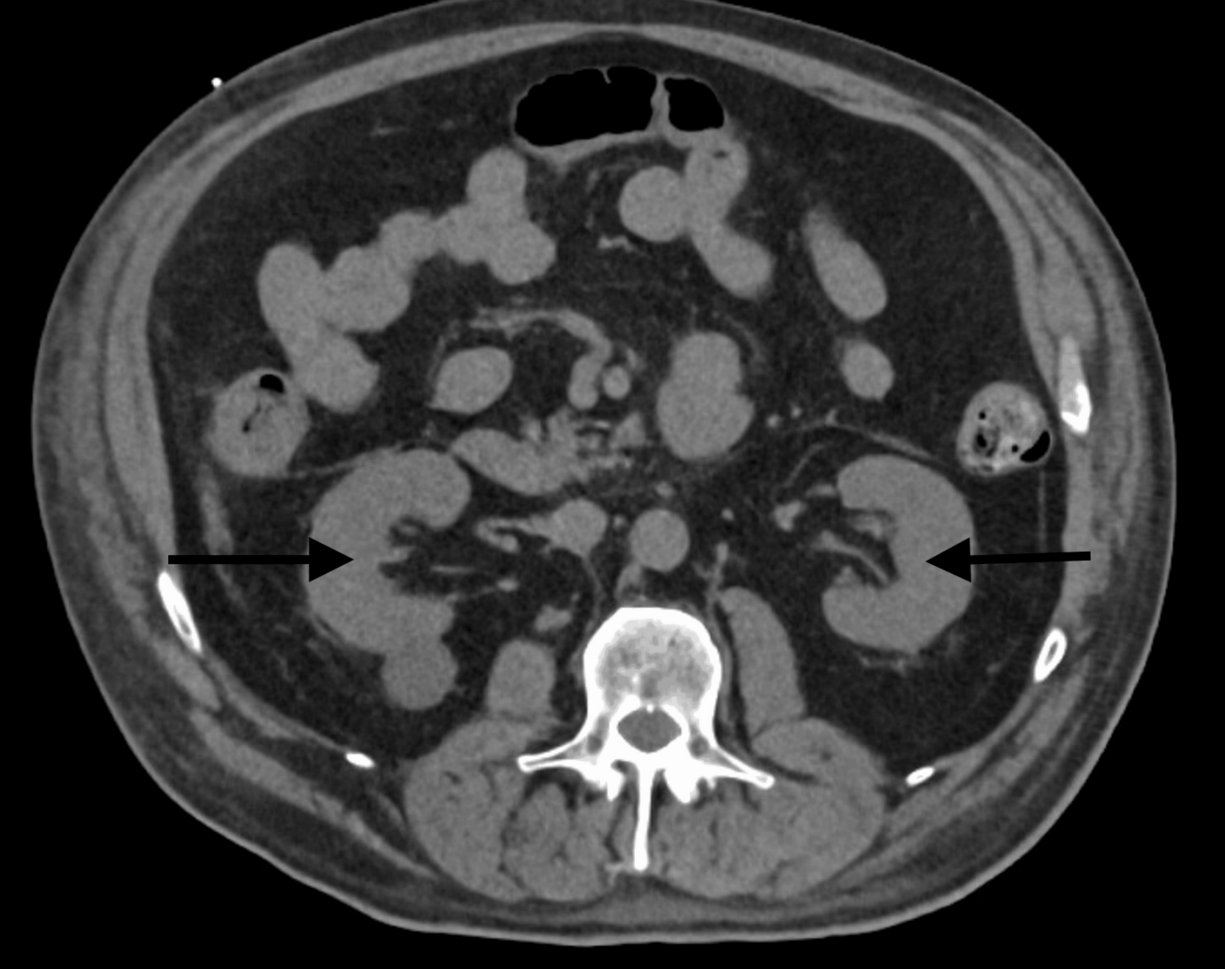
Axial Non-contrast CT of the Abdomen - Kidneys The CT image demonstrates both kidneys with preserved size and contour, without hydronephrosis, obstructing stones, or masses (black arrows).

**Figure 4:**
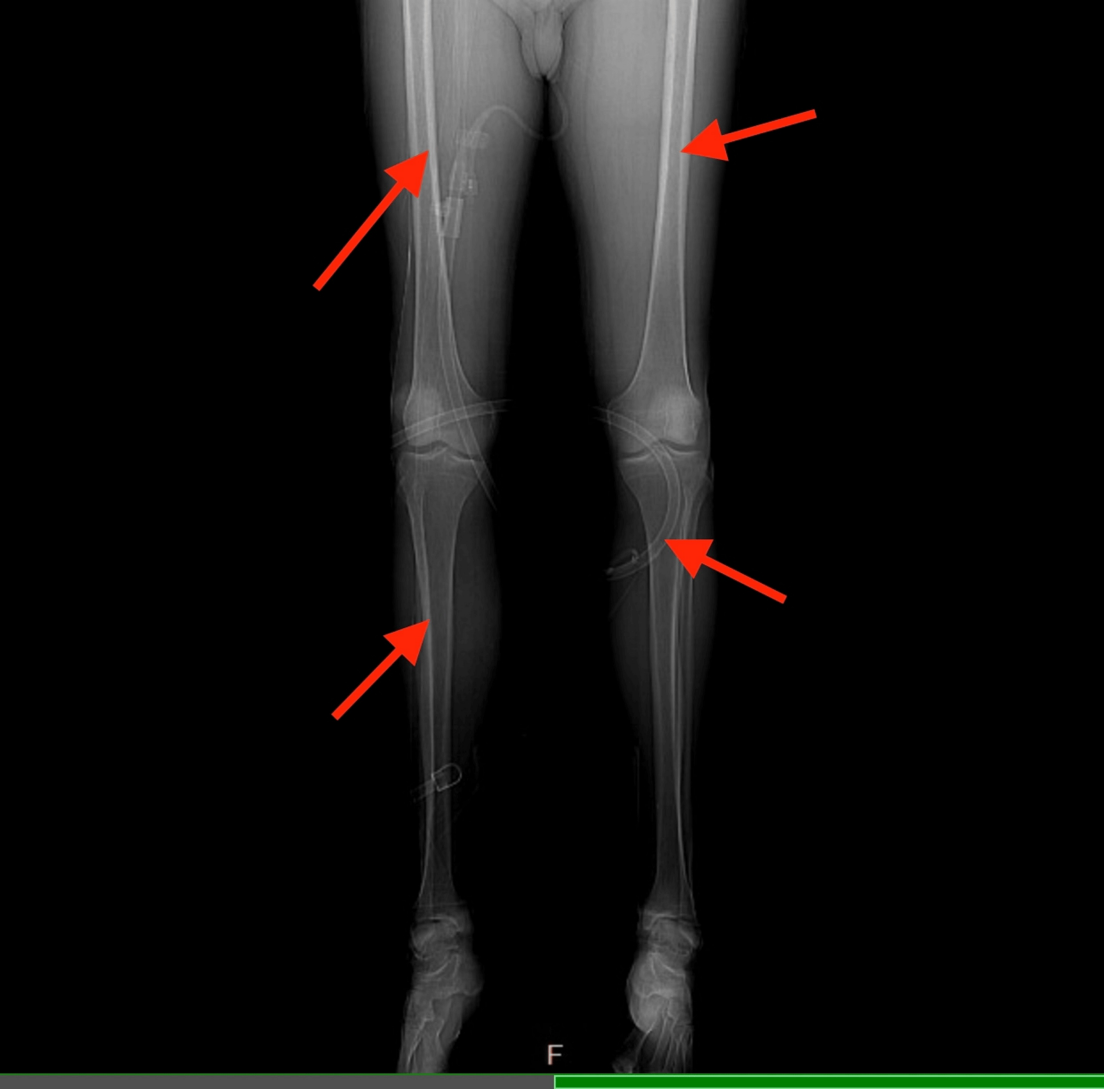
Frontal CT image of the lower extremities (bone window) shows no evidence of acute fractures, periosteal reaction, or destructive bony lesions (red arrows). Bone mineralization is preserved.

An MRI of the left thigh demonstrated muscular and subcutaneous edema without signs of abscess, myonecrosis, or compartment syndrome (Figure 1). Non-contrast CT imaging of the abdomen showed no obstructive or structural abnormalities of the liver (Figure 2), and no hydronephrosis, masses, or structural abnormalities of the kidneys (Figure 3). A CT of the lower extremities ruled out acute fractures (Figure 4).

A myositis panel, including anti-histidyl-tRNA synthetase antibody (anti-Jo-1), antinuclear antibody (ANA), anti-topoisomerase I antibody (anti-Scl-70), and anti-ribonucleoprotein antibody (anti-RNP), was negative. Thyroid function testing revealed profound hypothyroidism, with a thyroid-stimulating hormone (TSH) of 121.7 µIU/mL and a free T4 level of less than 0.4 ng/dL. Atorvastatin was promptly discontinued, and levothyroxine was increased to 150 mcg daily.

Despite supportive measures, the patient developed progressive volume overload with respiratory compromise, necessitating initiation of hemodialysis on hospital day 3. By hospital day 6, his leg pain began improving, and liver enzymes showed a downward trend. By day 10, his CK level began to decline.

His laboratory parameters gradually improved with continued management. CK trended down to 17,487 U/L by April 28, 8,065 U/L by April 30, and 5,072 U/L by May 2. Creatinine decreased from a peak of 11.0 mg/dL to 6.4 mg/dL, and BUN improved from 126 to 64 mg/dL. Transaminases also declined steadily. These trends are summarized in Table 2. This biochemical improvement mirrored the patient’s gradual clinical stabilization.

**Table 2:** Temporal Trends in Creatine Kinase, Renal Function, and Liver Enzymes CK: creatine kinase; BUN: blood urea nitrogen; AST: aspartate aminotransferase; ALT: alanine aminotransferase; (H): high; (HH): critically high; N/A: not available

Date	CK (U/L)	Creatinine (mg/dL)	BUN (mg/dL)	AST (U/L)	ALT (U/L)
04/17/25	>20,000 (HH)	9.6 (H)	84 (H)	968 (H)	498 (H)
04/18/25	>20,000 (HH)	9.9 (H)	86 (H)	1161 (H)	495 (H)
04/19/25	>20,000 (HH)	7.2-7.3 (H)	50-51 (H)	1304 (H)	557 (H)
04/20/25	>20,000 (HH)	8.1 (H)	60 (H)	N/A	N/A
04/21/25	>20,000 (HH)	9.6 (H)	72 (H)	1501 (H)	677 (H)
04/23/25	>20,000 (HH)	8.7 (H)	77 (H)	808 (H)	549 (H)
04/25/25	>20,000 (HH)	7.9 (H)	73 (H)	446 (H)	390 (H)
04/28/25	17,487 (HH)	11.0 (HH)	126 (HH)	N/A	N/A
04/30/25	8,065 (HH)	8.0 (H)	75 (H)	174 (H)	185 (H)
05/02/25	5,072 (HH)	6.4 (H)	64 (H)	116 (H)	166 (H)

The patient was ultimately discharged to an acute rehabilitation facility with plans for outpatient hemodialysis and close endocrine follow-up. This case illustrates the importance of considering thyroid function in patients with unexplained rhabdomyolysis, particularly when recent statin use and atypical biochemical findings are present.

## Discussion

Statins are cornerstone therapies for cardiovascular prevention but carry a rare risk of rhabdomyolysis, particularly in predisposed patients. The estimated incidence of statin-induced rhabdomyolysis ranges from 0.01% to 0.1%, based on population-level analyses of hospitalized cases [1]. The risk may be significantly increased in the presence of severe hypothyroidism, a condition that alters drug metabolism, muscle energetics, and repair mechanisms.

In this case, a 76-year-old male developed excruciating lower extremity pain, elevated CK >25,000 U/L, transaminitis, and anuric AKI shortly after initiating atorvastatin. Further workup revealed profoundly uncontrolled hypothyroidism, with TSH >120 µIU/mL and free T4 <0.4 ng/dL. This constellation is consistent with prior reports of statin-induced rhabdomyolysis potentiated by hypothyroidism [2-4]. The proposed mechanisms include impaired mitochondrial adenosine triphosphate (ATP) production in muscle cells, increased membrane fragility, and reduced hepatic metabolism of statins, potentially via downregulation of cytochrome P450 activity [5].

Although not a universal finding, subclinical or undiagnosed hypothyroidism has repeatedly emerged as a trigger for statin myopathy [3,4,6]. Our patient’s delayed CK decline, which remained >25,000 U/L for over 10 days, also parallels published cases showing prolonged muscle injury and biochemical recovery when thyroid dysfunction is present [7,8].

Initial concern for hepatic injury was raised by marked AST and ALT elevations, but normal bilirubin levels, imaging without hepatobiliary pathology, and persistent muscle pain suggested muscle-derived transaminases. This distinction is critical to avoid misdiagnosis and has been emphasized in other statin-related rhabdomyolysis cases [9,10].

The patient progressed to dialysis-dependent AKI, a well-documented outcome of severe rhabdomyolysis caused by myoglobin-induced acute tubular necrosis, as shown in multiple case reports of statin-induced muscle injury in the hypothyroid population [5,6,11]. Some have theorized that hypothyroidism may further reduce renal perfusion, compounding renal injury, but this remains speculative and is not directly confirmed by current literature.

Compartment syndrome was considered based on the patient’s persistent pain and MRI findings of significant muscular edema. Though ultimately excluded, this rare complication should remain on the differential, particularly in severe rhabdomyolysis. Dunphy et al. described a similar case triggered by exertion and statin use, highlighting that physical activity often plays a larger role in such outcomes [9,12].

Although drug interactions, particularly with agents like gemfibrozil, are known contributors to statin toxicity, our patient was not on any interacting agents. Nonetheless, Dalugama et al. emphasized the importance of thorough medication reconciliation to avoid synergistic toxicity [10,13].

This case highlights the importance of evaluating underlying physiologic vulnerabilities before prescribing statins. Although Graham et al. [12] supported general risk-factor screening to reduce rhabdomyolysis risk, it does not specifically recommend TSH or free T4 testing. However, based on mounting evidence from case reports [2-4,6,11], clinicians should consider thyroid function testing in older adults or those with a known or suspected thyroid history before starting statins. Doing so may prevent life-threatening complications like those seen here.

## Conclusions

This case highlights the potentially life-threatening interaction between statin therapy and severe hypothyroidism, resulting in dialysis-dependent AKI and prolonged hospitalization. Undiagnosed or undertreated thyroid dysfunction can significantly increase the risk of statin-induced rhabdomyolysis. While routine thyroid function screening is not currently standard practice, this case and others suggest it may be justified in older adults or those with known or suspected thyroid disease to help prevent avoidable complications.
